# Heterochromatin components in germline stem cell maintenance

**DOI:** 10.1038/srep17463

**Published:** 2015-12-02

**Authors:** Yalan Xing, Willis X. Li

**Affiliations:** 1 Department of Biomedical Genetics, University of Rochester Medical Center, Rochester, NY 14620; 2 Department of Medicine, University of California San Diego, La Jolla, CA 92093

## Abstract

Stem cell maintenance requires expression of genes essential for stemness and repression of differentiation genes. How this is achieved remains incompletely understood. Here we investigate the requirement for central components of heterochromatin, Heterochromatin Protein 1 (HP1) and the histone H3 lys9 methyltransferase Su(var)3-9, in the *Drosophila* male germline stem cell (GSC) self-renewal, a paradigm for studying adult stem cell behavior. We found that mutations or RNAi knock down of *HP1* or *Su(var)3-9* cause loss of GSCs, accompanied by defects in cell division or survival and premature expression of the differentiation gene *bag of marbles* (*bam*). Conversely, over-expressing HP1 increases GSC number in wildtype flies and, strikingly, restores fertility to the sterile *hopscotch* (*hop*) mutant flies that lack niche signals. These results suggest that the central components of heterochromatin play roles including repressing differentiation genes in *Drosophila* male GSC maintenance.

Stem cells possess unique epigenetic modifications and gene expression profiles that are conducive to their function: long-term maintenance of undifferentiated state yet poised for differentiation^1^,^2^,^3^. A salient feature of stem cells is that many developmentally important genes are epigenetically repressed. For instance, it has been shown that the Polycomb group proteins (PcGs) confer repressive chromatin modifications and are essential for maintenance of both embryonic and adult stem cells^4^,^5^,^6^. The molecular mechanisms controlling stem cells self-renewal are not completely understood, and different types of stem cells may use different strategies to repress differentiation genes. Heterochromatin formation, marked by histone H3 lys9 di- or tri-methylation (H3K9me2,3), is responsible for epigenetic gene repression in many developmental contexts^7^,^8^. Central components of heterochromatin comprise Heterochromatin Protein 1 (HP1) and histone H3 lys9 methyltransferases, including Su(var)3-9 and SETDB1^7^,^8^. It has been reported that SETDB1 and its *Drosophila* homolog Eggless (Egg or dSETDB1) are essential for maintaining self-renewal of embryonic stem cells in mice and of adult germline stem cells in *Drosophila*, respectively^9^,^10^. In the *Drosophila* ovary, it has been shown that dSETDB1 and Su(var)3-9 sequentially function during GSC differentiation^11^. In planarians upon injury, HP1 is expressed and promotes self-renewal and triggers proliferation of adult stem cells for tissue regeneration^12^. However, it remains to be established that heterochromatin formation participates in stem cell self-renewal.

The male reproductive system in *Drosophila* provides an excellent model for understanding the fundamental mechanisms underlying stem cell regulation^1^,^2^,^13^ (Fig. S1A). At the apex of the testis, a group of post-mitotic somatic cells called hub cells comprise a key component of the male GSC niche, maintaining 8 to 12 germline stem cells (GSCs)^14^. GSCs and their primary derivatives, gonialblasts (GBs) and spermatogonia, express the germline specific protein Vasa; they are located adjacent to their niche – the hub cells, which express Fasciclin III (FasIII)^1^,^2^,^13^. GSCs are attached to the hub cells via adherens junctions. Hub cells express the cytokine-like ligand Unpaired (Upd), which activates the JAK/STAT (Hopscotch/STAT92E) pathway in the GSCs as well as the somatic cyst stem cells (CySCs), instructing their self-renewal. Upon dividing asymmetrically, a GSC produces two daughter cells. One of them retains contact with the hub and maintains stem cell identity, while the other is displaced from the hub to become a gonialblast, which begins transit-amplifying divisions as spermatogonia. Spermatogonia go through four synchronous mitotic divisions, resulting in 16 spermatocytes. Without JAK/STAT signaling, GSCs differentiate but do not self-renew, while ectopic JAK/STAT signaling greatly expands the stem cell population^15^,^16^. Further studies have indicated that JAK/STAT signaling primarily regulates self-renewal of somatic CySCs, which are essential for GSC self-renewal^17^,^18^. Despite these findings, the precise mechanisms controlling stem cell self-renewal remain incompletely understood.

Here, we investigated whether heterochromatin components are required for the maintenance of *Drosophila* male GSCs and examined the effects of overexpression or reduction of HP1 or Su(var)3-9, central heterochromatin components, on GSC numbers in *Drosophila* testes. Our results indicate that both HP1 or Su(var)3-9 are important for GSC self-renewal and for repressing differentiation genes, such as *bam of marbles* (*bam*), although they may also play general roles such as cell division and survival. Despite these general functions, our results suggest that proper heterochromatin formation might have a specific role in epigenetically repressing the expression of differentiation genes in GSCs, which is important for maintaining their self-renewal.

## Results

### Role of Heterochromatin components in GSC maintenance

To investigate the role of central heterochromatin components HP1 and Su(var)3-9 in GSC maintenance, we used genetic mosaics or RNAi-mediated knock down to determine the effects of loss of HP1 or Su(var)3-9 on GSCs. Heterochromatin Protein 1 (HP1) and the histone H3 lys9-specific methyltransferase Su(var)3-9 are central for heterochromatin formation, and altering HP1 or Su(var)3-9 levels directly impacts heterochromatin formation^7^. To identify GSCs, we carried out immunostaining with anti-alpha-spectrin antibodies to identify fusomes, with antibodies against the cell surface marker FasIII to mark hub cells (stromal cells), and with anti-Vasa antibodies to identify germ cells. GSCs can be identified as Vasa+ cells that are adjacent to the hub and that contain a dotted fusome.

We first generated clonal cells homozygous for a *Su(var)3-9* null mutation, *Su(var)3-9*^*2*^, using FLP/FRT-mediated mitotic recombination with a GFP marker, such that mutant cells can be identified as GFP^–^ cells (see Methods). *Su(var)3-9*^*2*^ is associated with a point mutation that abolishes the HMTase catalytic activity^19^. *Su(var)3-9*^*2*^ homozygous flies were not viable, but became fully viable when a *hsp70-Su(var)3-9*^+^ transgene was expressed in the background (n > 100), ruling out the possibility that the *Su(var)3-9*^*2*^ chromosome carried an unrelated lethal mutation. We thus used the *Su(var)3-9*^*2*^ chromosome to carry out clonal analysis.

**Figure 1:**
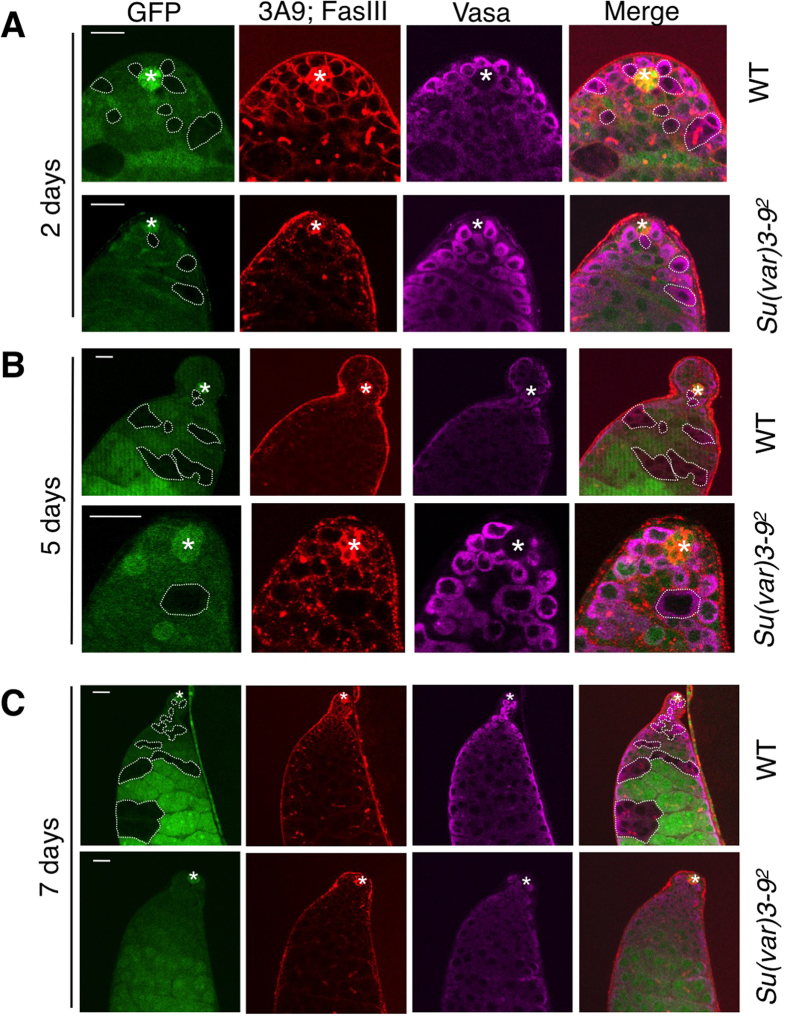
Effects of *Su(var)3-9* mutation on GSC maintenance. Testes from male flies that had been subjected to induction of marked clones for loss-of-function of *Su(var)3-9* were immunostained for Vasa (magenta cytoplasmic staining) and for fusomes and hub cells (both red). Scale bar = 20 μm. Testes were dissected after 2 **(A)**, 5 **(B)**, or 7 **(C)** days after clone induction. Wild-type control and *Su(var)3-9*^*2*^ homozygous mutant clonal cells were marked by the absence of GFP (lack of green; circled by dotted lines). Note that wild-type control clones were always found at GSC positions (next to the hub) and as differentiated spermatogonia, and that *Su(var)3-9*^*2*^ homozygous mutant clones were found at the GSC position only 2 day after clone induction, but not after 5 or 7 days, although GFP^–^ spermatogonia were still found. Also note the increased nuclear size of the *Su(var)3-9*^*2*^ homozygous cell (GFP^–^) in 5 days after clone induction.

When examined 2 days after clone induction, wild-type control GSC, GB, spermatogonial clones were frequently found; clones of *Su(var)3-9*^*2*^ homozygous GSCs, GBs, and spermatogonia were also found, albeit at a lower frequency (Fig. 1A; Table 1). However, when examined 5 and 7 days after clone induction, whereas wild-type control clones were frequently identified that included GSCs, GBs, spermatogonia, and spermatocytes, *Su(var)3-9*^*2*^ clones were found at much lower frequency and usually not as GSCs but as spermatocytes only (Fig. 1B,C; Table 1), suggesting that *Su(var)3-9*^*2*^ cells cannot remain as GSCs but can still undergo differentiation to give rise to spermatocytes. Consistent with the Su(var)3-9’s function as an H3K9 methyltransferase in heterochromatin formation and chromosomal compaction, *Su(var)3-9*^*2*^ mutant cells exhibited increased nuclear size (Fig. 1B), suggesting a loss of chromosomal compaction, and a loss of the heterochromatin marker H3K9me3 (Fig. S1D). Interestingly, *Su(var)3-9*^*2*^ mutant clones (Fig. 1B), as well as *Su(var)3-9* RNAi-expressing clones (see below) contained fewer than 16 cells/cyst, suggesting that Su(var)3-9 may also play a role in spermatocyte division or differentiation.

To confirm the role of Su(var)3-9 in GSC maintenance, we tested additional strong or null alleles, *Su(var)3-9*^*17*^ and *Su(var)3-9*^*6*^^19^. Testes from *Su(var)3-9*^*17/6*^transheterozygous male survivors had no detectable heterochromatin marker H3K9me3 signal and were thinner (Fig. S1C). In addition, *Su(var)3-9*^*17/6*^transheterozygous males lost their GSCs more precipitately than controls. By counting Vasa+ GSCs in 1- and 30-day-old males, we found that while 1- and 30-day-old control males had on average 7.9 ± 1.1 and 5.7 ± 1.2 GSCs/testis, respectively, *Su(var)3-9*^*17/6*^ males had 7.4 ± 1.7 and 3.6 ± 1.3 GSCs/testis, respectively. 30-day-old *Su(var)3-9*^*17/6*^ males had significantly fewer GSCs than their control siblings (p = 0.006; Student’s *t*-Test). These observations are consistent with the idea that Su(var)3-9 is important for male GSC maintenance.

**Figure 2:**
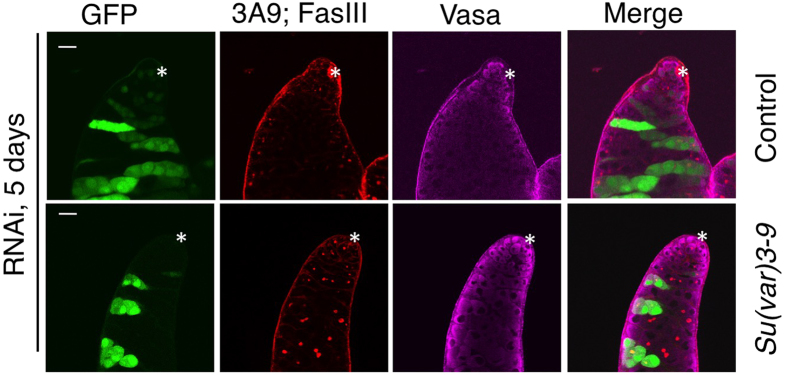
Effects of knocking down *Su(var)3-9* by RNAi on GSC maintenance. Testes from male flies expressing a *Su(var)3-9* RNAi transgene with the germline specific *nanos-Gal4* were immunostained for Vasa (magenta cytoplasmic staining) and for fusomes and hub cells (both red). Asterisk marks the position of the hub. Scale bar = 20 μm. In control testes (which did not express *Su(var)3-9* RNAi) 5 days after clone induction, clonal GFP+ cells were found as both GSCs (next to the hub) and spermatogonia. Five days after *Su(var)3-9* RNAi was expressed, no GFP+ GSCs were observed next to the hub, although GFP+ spermatogonia were still observed.

As an independent test for the requirement of Su(var)3-9 in GSC maintenance, we expressed *Su(var)3-9* RNAi in random clonal cells that were marked by GFP (see Methods). We examined GFP+ cells 2, 5, and 7 days after clone induction. We found that while in the control experiment (no *Su(var)3-9* RNAi expression), GFP+ clonal cells were abundantly found as both GSCs and differentiated cysts, GFP+ cells expressing *Su(var)3-9* RNAi, however, were found as GSCs (at a lower frequency) only 2 and 5 days, but not 7 days after clone induction (Fig. 2; Table 1). Seven days after clone induction, GFP+ cells expressing *Su(var)3-9* RNAi were found only as differentiated cysts (Fig. 2; Table 1). Taken together, the above two types of clonal studies suggest that loss of Su(var)3-9 caused loss of GSCs, and may also cause defects in GSC division or differentiation.

We also carried out similar experiments with a null mutation and RNAi transgenes targeting *Su(var)205*, which encodes HP1. We found that, similarly to *Su(var)3-9*^*2*^ mutant clones, clones of *Su(var)205* null GSCs (*Su(var)205*^5^) were found two days after clone induction, albeit at a lower frequency than wild-type control clones (Table S1). In contrast to *Su(var)3-9*^*2*^clones, however, we never recovered any mature *Su(var)205*^5^ homozygous spermatocytes (Fig. S2A; Table S1), suggesting that complete loss of HP1 may cause lethality to GSCs. Similar results were obtained with expressing *Su(var)205* RNAi in random clones (Fig. S2B). Since HP1 is not only essential for heterochromatin formation, but also has heterochromatin-independent functions, complete loss of HP1 or heterochromatin may cause cell death. Nevertheless, the above genetic mosaic studies using *Su(var)3-9* and *Su(var)205* mutations and their RNAi transgenes suggest that the major heterochromatin components, HP1 and Su(var)3-9, are essential for GSC maintenance, possibly playing differential roles in survival, division, and differentiation.

### Role of HP1 and Su(var)3-9 in GSC-specific gene expression

**Figure 3:**
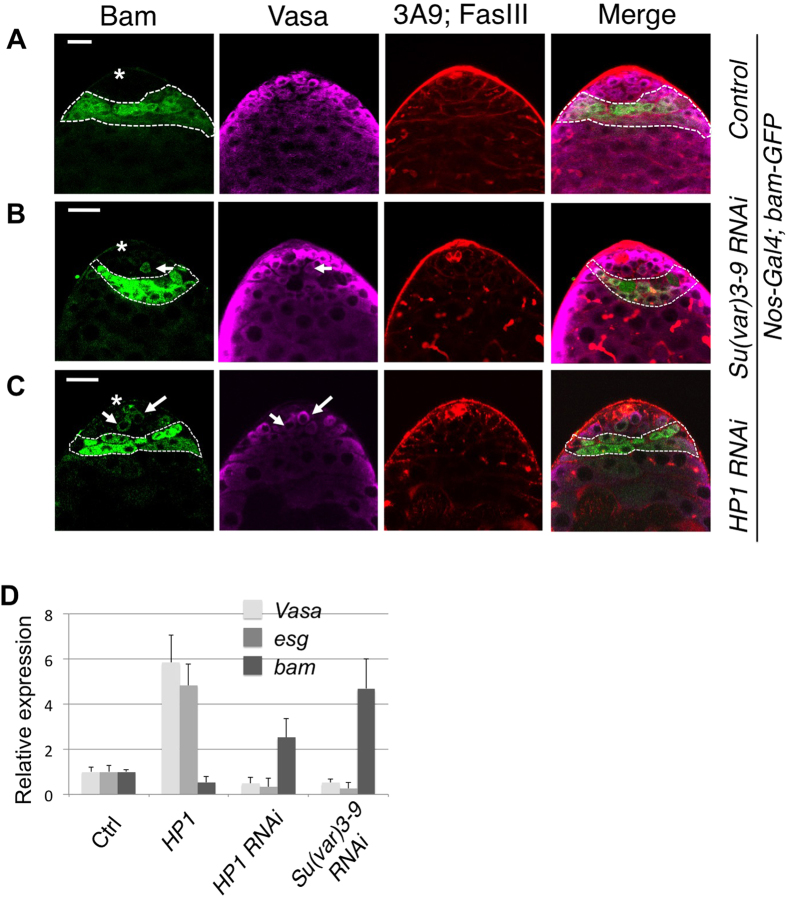
Loss of Su(var)3-9 and HP1 disrupts GSC specific gene expression. **(A-C)** Testes from male flies of indicated genotypes were immunostained with mAb3A9 and anti-FasIII (both red) to reveal the fusome and hub cells, respectively, and with anti-Vasa (magenta). Green shows *bam-GFP* expression. Brackets indicate the band of *bam-GFP* cells normally detected in differentiated GSCs. The asterisk marks the hub. Scale bar = 20 μm. **(A)** A wild-type control testis, in which *bam-GFP* is expressed in spermatogonia three cell diameters away from the hub and is not expressed in GSCs and gonialblasts. **(B)** In a testis expressing *Su(var)3-9* RNAi, *bam-GFP* is detected in cells next to the hub and also in those in gonialblast positions. The arrow points to one such cells. **(C)** In a testis expressing *HP1* RNAi, *bam-GFP* is detected in cells in gonialblast positions and also weakly in cells next to the hub (arrows). The arrowhead points to a spermatogonia that expresses *bam-GFP* but has lost Vasa expression. **(D)** Individual testes of indicated genotypes were dissected and the tip region (see Methods) was used for qPCR experiments to quantify mRNA levels of *rp49* (control), *esg*, *Vasa*, and *bam*. The results were normalized to *rp49* and then to wild-type testis. Error bars represent standard deviations of three independent experiments.

To understand whether HP1 and Su(var)3-9 play specific roles in GSC maintenance in addition to their general functions in cell division and survival, we examined changes in expression of a few known genes that are differentially expressed in GSCs. The differentiation marker gene *bag of marbles* (*bam*)^20^,^21^ is normally expressed in 4–16 cell differentiated spermatogonia cysts but not in GSCs and their immediate progeny, gonialblasts, resulting in a band of *bam* expression three cell diameters away from the hub^20^, as revealed by a *bam-GFP* reporter^17^ (Fig. 3A). We found that knocking down *Su(var)3-9* by RNAi, driven by the germline specific *nanos-Gal4*, resulted in ectopic expression of *bam-GFP* in gonialblasts, as judged by the location of cell ectopically expressing *bam-GFP* (Fig. 3B), suggesting that these cells had undergone premature differentiation. *HP1 RNAi* knock-down also resulted in ectopic expression of *bam-GFP* in GSCs and gonialblasts, as cells immediately adjacent to the hub now expressed *bam-GFP* (Fig. 3C). In addition, knocking-down HP1 caused loss of the germline-specific marker Vasa in a fraction of germline cells, although some of these spermatogonia maintained *bam-GFP* expression (Fig. 3C, arrow). These results suggest that HP1 and Su(var)3-9 might be important for maintaining the unique expression profiles of GSCs and their progeny.

To further investigate the role of HP1 and Su(var)3-9 in GSC gene expression, we performed qPCR experiments using testis tip regions (see Methods) to determine changes in expression of genes that are differentially expressed in GSCs. We found that HP1 over-expression caused an increase in the expression levels of germline or GSC-specific genes, *esg* and *Vasa*, and a decrease in those of the differentiation gene *bam*, while RNAi knockdown of HP1 and Su(var)3-9 had the opposite effects (Fig. 3D). Thus, heterochromatin components HP1 and Su(var)3-9 might be important for regulating differential expression of genes that are essential for GSC maintenance.

### HP1 overexpression rescues GSC loss mutant phenotype

Next, we investigated whether the heterochromatin components HP1 and Su(var)3-9, when overexpressed, can promote GSC self-renewal. It has been shown that GSC self-renewal requires activation of JAK/STAT signaling by Upd secreted from the hub^15^,^16^. We investigated whether over-expressing HP1 could restore GSCs to the *hop*^*25*^ male survivors that do not have GSCs.

**Figure 4:**
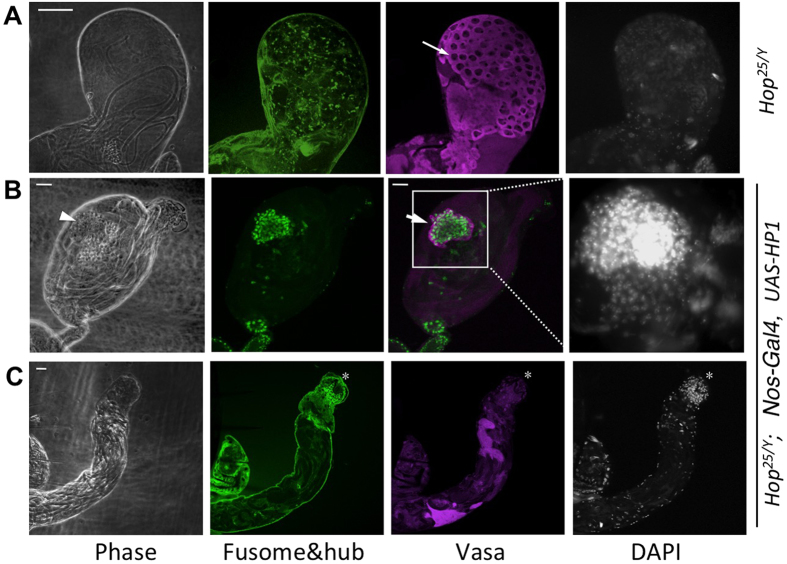
HP1 overexpression rescues GSC loss mutants. Testes from 3-day old *hop*^*25*^*/Y* male survivors of indicated genetic backgrounds were stained with DAPI, anti-Vasa, mAb3A9 and anti-FasIII, as indicated. **(A)** A *hop*^*25*^*/Y; nos-Gal4* testis showing no sperm bundles, with all Vasa-positive germ cells exhibiting large nuclei (arrow) and dotted fusome but lacking intense DAPI signals. **(B)** A partially rescued *hop*^*25*^*/Y; nos-Gal4/UAS-HP1* testis. Thick sperm bundles were seen (left). A fraction of Vasa-positive germ cells contain small DAPI-dense nuclei, dotted fusome, resembling GSCs (arrow), were observed. **(C)** A completely rescued testis from a *hop*^*25*^*/Y; nos-Gal4/UAS-HP1* male survivor that had become fertile. The testis resembles that of wild-type flies, with mature long sperm bundles.

*hop*^*25*^ is a hypomorphic allele of *hop*; *hop*^*25*^ hemizygous males usually die, but occasionally escapers can be found that are completely sterile, with their testes lacking GSCs^15^,^16^. Indeed, testes of *hop*^*25*^ hemizygous male survivors lack *esg-GFP*^*+*^ cells or any small cells with condensed nuclei that are characteristic of GSCs and that can be distinguished by intense DAPI staining (Fig. 4A). Upon staining with antibodies that distinguish different cell types in the testis, we found that testes from *hop*^*25*^ hemizygous males contain exclusively large Vasa-positive germ cells (Fig. 4A, arrow). This is in contrast to a previous report that *hop*^*25*^ hemizygous testes had no germ cells^16^. In wild-type testes, large Vasa-positive germ cells contain branched fusomes. However, the large germ cells in *hop*^*25*^ hemizygous testes contain only dotted (not branched) fusomes (Fig. 4A) and were negative for the differentiation marker *bam-GFP*. We suggest that the large Vasa-positive (*bam*-negative) cells in *hop*^*25*^ hemizygous testes might represent primordial germ cells (PGCs) that were not induced to form GSCs due to lack of JAK/STAT signaling. Nonetheless, a few elongated spermatids were found in *hop*^*25*^ hemizygous testes, which could be derived from a few rare “escaper” GSCs in these testes (Fig. 4A, left), although *hop*^*25*^ hemizygous males are sterile.

We expressed HP1 in *hop*^*25*^ hemizygous males using the *nanos-Gal4* driver and examined their effects on the *hop*^*25*^ testes. We found that expressing HP1 rescued, to some extent, GSC formation in *hop*^25^ hemizygous testes, i.e., cells resembling GSCs were detected as small Vasa-positive cells that were brightly stained with DAPI in the testes (Fig. 4B). Consistent with a partial rescue of GSC formation, more thick bundles of differentiated spermatids were observed in these testes (Fig. 4B, left).

Strikingly, expressing HP1 was able to restore fertility to the otherwise sterile *hop*^*25*^ hemizygous males, such that a few *hop*^*25*^*/Y; nosGal4/UAS-HP1* males sired progeny when crossed to wild-type virgin females (n = 4/13), while none of the *hop*^*25*^*/Y; nosGal4/CyO* control sibling males became fertile (n = 15). The presence of the *hop*^*25*^ and *UAS-HP1* chromosomes in the rescued males was confirmed in the F1 generation. Without the HP1 transgene expression, no fertile *hop*^*25*^*, nosGal4/Y* male flies were ever found (n > 100; also see^15^,^16^). We dissected the fertile *hop*^*25*^*/Y; nosGal4/UAS-HP1* males and found that, interestingly, some of them had asymmetric testes, with one testis having an elongated morphology containing a few sperm bundles (Fig. 4C), while the other retaining the “bulb” appearance of the typical *hop*^*25*^ hemizygous testis (see Fig. 4B). All the *hop*^*25*^*/Y; nosGal4/CyO* control sibling males examined had both testes exhibiting the “bulb” phenotype. Thus, over-expressing HP1 may promote GSC development or self-renewal even in the absence of adequate niche signals from the hub, enough for restoring fertility to the sterile *hop*^*25*^ hemizygous males.

### Levels of heterochromatin components influence GSC number

**Figure 5:**
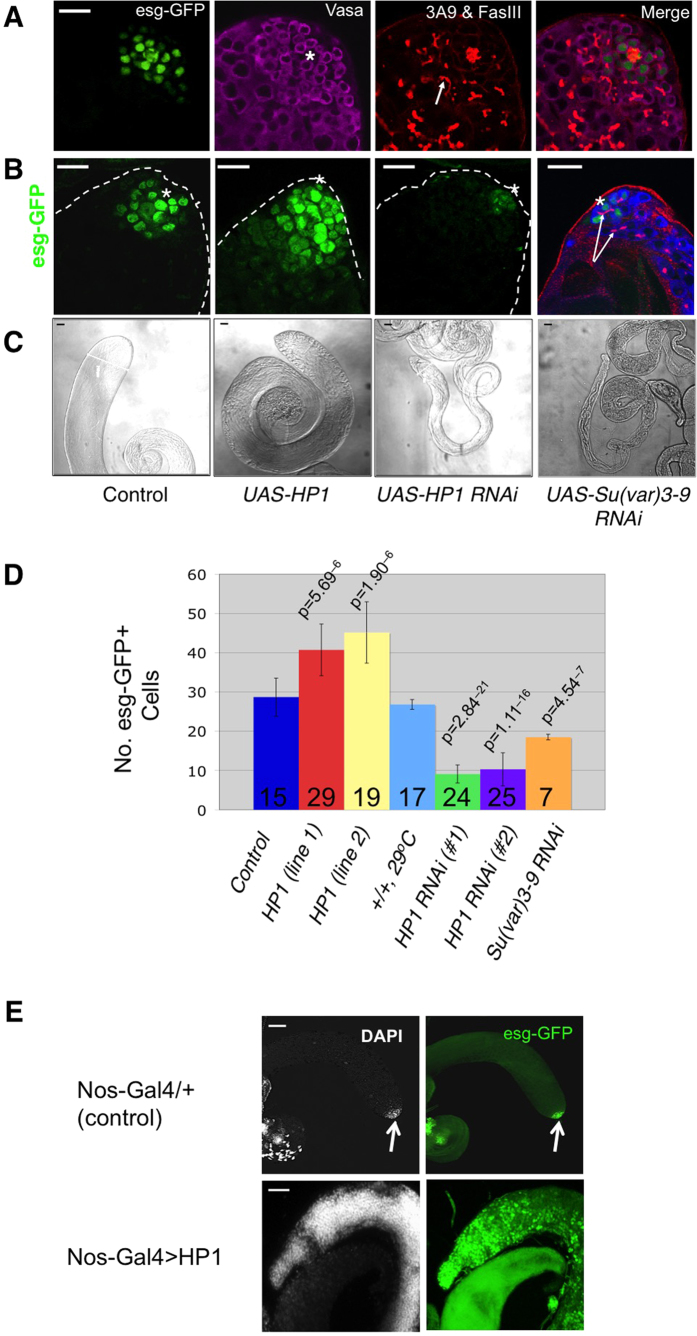
Levels of HP1 and Su(var)3-9 affect GSC number. Scale bar = 20 μm. **(A)** The apex of a control testis expressing *esg-GFP*. Testes were immunostained with mAb3A9 and anti-FasIII (both red) to reveal the fusome and hub cells, respectively. GFP fluorescence (green) is detected in GSCs and GBs, as well as hub cells, but not in the somatic CySCs or CCs. The hub is marked with an asterisk. Germ cells (including GSCs) are positve for Vasa (magenta), which is a cytoplasmic protein specifically expressed in germ cells. GSCs usually contain spherical fusomes (red dots), and differentiated germline cysts are marked by the presence of branched fusomes (arrow). Scale bar = 20 μm. **(B)** Testis apex (outlined by dotted line) from 3-day old male flies expressing *esg-GFP* (green) and indicated transgenes (bottom) driven by *nos-Gal4*. The hub is marked with an asterisk. Branched fusomes are indicated (mAb3A9, red, arrows). **(C)** Effects of expressing indicated transgenes using *nanos-Gal4* on the morphology of the testis. Representative images from 3-day old male flies are shown at the same scale. Note the HP1 or Su(var)3-9 knockdown testes have a smaller diameter than the wild-type control (left). **(D)** Quantification of esg-GFP+ cells of testes expressing indicated transgenes driven by *nanos-Gal4*. Numbers of testes analyzed are indicated; p: significance compared to control by Student’s *T*-test. **(E)** Testes were dissected from 40-day old adult *nos-Gal4/+*; *esg-GFP/UAS-HP1* or control (*nos-Gal4/+*; *esg-GFP/+*) males and were scanned for GFP and DAPI. Note that the wild-type control testis has DAPI-dense and GFP+ cells present only at the tip region, while the *nos-Gal4* > *HP1* testis exhibits great expansion of esg-GFP+ and DAPI-dense cells.

To further substantiate that HP1 and Su(var)3-9 are important for GSC maintenance, we tested the effects of altering their levels on *Drosophila* male GSC numbers. We used an *esg-GFP* enhancer trap line to estimate GSC number because it allows direct observation of GFP+ cells in dissected testes without immunostaining. *esg-GFP* is inserted in the *escargot* (*esg*) locus (*esg*-GFP; see Methods) and expresses high levels of GFP specifically in GSCs and gonialblasts (GBs), but not in CySCs (Fig. 5A, S3). *esg* encodes a transcription factor important for GSC function^22^,^23^, and is specifically expressed in many types of adult stem cells^24^,^25^,^26^. Although *esg-GFP* is also expressed in hub cells, these cells are clearly distinguishable from GSCs and GBs due to their morphology and unique localization (Fig. 5A). Although *esg* might also be expressed in CySCs, the *esg*-GFP used in this study was not detectable in CySCs (Fig. S3). An *esg-lacZ* enhancer trap line has previously been used to mark *Drosophila* male GSCs and GBs^27^. Therefore, GFP+ cells in the testis of *esg-GFP* males can be used to estimate the abundance of GSCs.

To confirm that *esg*-GFP can be used for estimating GSCs, we carried out immunostaining with anti-spectrin antibodies to identify fusomes, with antibodies against the cell surface marker FasIII to mark hub cells, and with anti-Vasa antibodies to identify germ cells (Fig. 5A). We found that, except for hub cells, all GFP-positive cells also co-expressed germline marker Vasa (Fig. 5A, S3), consistent with the idea that *esg*-GFP marks GSCs and GBs. By analyzing serial optical sections using confocal microscopy, we found that there are on average 26.8 ± 2.6 GFP^+^ cells (excluding hub cells; n = 27 testes) in the testis of wild-type males carrying a copy of *esg-GFP*, corresponding to the number of GSCs and their immediate progeny (gonialblasts) present in the testis.

To assess the effects of altering HP1 and Su(var)3-9 on GSCs, we used the *nanos-Gal4* driver to express transgenes in the germline, and examined 3-day old adult testes. We found that over-expressing HP1 caused an increase in the number of GFP^+^ cells, while expressing a *HP1-RNAi* or a *Su(var)3-9 RNAi* transgene dramatically reduced the GFP^+^ cell population and resulted in appearances of branched fusomes near the hub (Fig. 5B**, arrows)**, and in shrinkage of the testis (Fig. 5C). In control and UAS-HP1 testes, GFP^+^ cells had dotted fusomes (not shown). These results support the idea that levels of HP1 and Su(var)3-9 influence GSC numbers.

To further determine to what extent HP1 overexpression can increase GSC number, we prolonged HP1 over-expression and examined the testes from adults 40 days after eclosion. We found that over-expressing HP1 in these conditions caused a great expansion of GSCs, such that the testis was, strikingly, filled with DAPI-dense, Vasa-positive GSCs and lacked any differentiated, elongated spermatid bundles (Fig. 5E). It has been previously reported that GSCs and their immediate daughter cells, gonialblasts (GBs), as well as Hub cells and CySCs contain highly condensed DNA, which is brightly stained with the DNA dye DAPI^16^,^27^. Wild-type control flies raised in parallel did not show over-population of GSCs, but rather exhibited reduced GSC number (Fig. 5E), consistent with previously reported GSC loss with aging^28^. These results suggest that HP1 over-expression promote GSC formation or maintenance, consistent with the above observation that over-expressing HP1 restored GSCs as well as fertility to *hop*^*25*^sterile flies that normally lack GSCs.

## Discussion

Global epigenetic transcriptional repression, especially of differentiation genes, is a common feature of all stem cells. Heterochromatin formation has long been known as an important epigenetic gene repression mechanism^7^. However, the role of heterochromatin formation in stem cell self-renewal has not been systematically studied. To understand the molecular mechanisms that control stem cell self-renewal, we have genetically investigated the roles of two major heterochromatin components, HP1 and Su(var)3-9, in *Drosophila* male GSC self-renewal. We have shown that HP1 and Su(var)3-9 are each necessary for GSC maintenance, and that HP1 is sufficient for GSC self-renewal in certain contexts in the *Drosophila* testis.

We have examined the effects of mutations or RNAi knock down of *HP1* or *Su(var)3-9* on *Drosophila* male GSCs, and have found in each case there was loss of GSCs, suggesting that *HP1* and *Su(var)3-9* are each essential for GSC maintenance. Loss of GSC could result from defects in cell division, self-renewal, or survival. The phenotypes of mutant GSCs indicate that HP1 might be required for all the processes, whereas Su(var)3-9 seems to be required for GSC cell division and self-renewal, but not survival. This is not surprising since HP1 and Su(var)3-9 are required for maintenance of constitutive heterochromatin, which is essential for chromosomal compaction of genome stability^7^,^8^. A role of HP1 and Su(var)3-9 in GSC self-renewal is supported by the finding that knocking down HP1 or Su(var)3-9 in GSC causes premature expression of the differentiation marker *bam* (Fig. 3). More importantly, we found that over-expression of HP1 promotes GSC proliferation and is sufficient for restoring GSC to mutant flies that lack GCSs. This property of HP1 is reminiscent of planarian HP1, which is induced upon injury to promote regenerative proliferation of adult stem cells^12^.

Previous work has established that JAK/STAT signaling from hub cells is essential for maintenance of germline and somatic stem cells^15^,^16^,^17^,^18^. In somatic tissues, we have previously shown that JAK overactivation counteracts the functions of HP1 and Su(var)3-9 and reduces heterochromatin to promote tumorigenesis^29^. These findings appear paradoxical, as in GSCs JAK activation and HP1 both positively regulate proliferation. We suggest that GSCs might respond to JAK/STAT activation differently from somatic cells due to the presence of different levels of regulatory components. For instance, it has been shown that the JAK/STAT signaling inhibitor, Suppressor of Cytokine Signaling 36E (Socs36E) is required in somatic but not germline stem cells for self-renewal^30^. A possibility is that GSCs lack inhibitory molecules such as Socs36E and thus allows overproduction of STAT92E due to autoregulation^31^, leading to high levels of unphosphorylated STAT92E, which we have previously shown to promote heterochromatin formation^31^,^32^. In this scenario, HP1 and heterochromatin are downstream targets of JAK/STAT signaling. Consistent with this idea, we have found that HP1 overexpression can substitute for JAK signaling in GSC generation or maintenance and restore fertility to sterile *hop* mutant males. The precise mechanisms by which JAK signaling regulates heterochromatin for GSC maintenance await further investigation.

Since HP1 and Su(var)3-9 are central components of heterochromatin, which plays global roles in cellular functions including gene repression and chromosomal compaction, it is possible that the observed effects of HP1 or Su(var)3-9 on GSCs are indirect. Thus mutations in HP1 or Su(var)3-9 may affect expression of a large number of genes, indirectly causing derepression of differentiation genes, such as *bam*. While how *bam* is repressed in GSCs requires further investigation, we think it is plausible that heterochromatin formation contributes to repression of differentiation genes in GSCs. This idea is consistent with the finding that overexpression of HP1, which promotes heterochromatin formation, increases GSC number (this study and^12^) and restores GSC to mutant flies that lack niche signals.

Genes important for heterochromatin formation or epigenetic repression have been implicated in stem cell maintenance. It has previously been shown that another H3K9 specific methylatransferase SETDB1, and its *Drosophila* homolog Eggless (Egg or dSETDB1), is essential for maintaining self-renewal of embryonic stem cells in mice and of adult germline stem cells in *Drosophila*, respectively^9^,^10^. In line with our findings, it has been shown that planarian HP1 promotes self-renewal and triggers regenerative proliferation of adult stem cells upon injury^12^. In addition, it has been shown that during *Drosophila* oogenesis, the DNA-associated protein Stonewall (Stwl) is required for GSC maintenance possibly by heterochromatin-mediated epigenetic repression of differentiation genes^33^, and that constitutive DNA methylation, another epigenetic gene repression mechanism often associated with heterochromatin formation, is essential for mouse hematopoietic stem cell self-renewal^34^. Taken together, these reports suggest that global epigenetic gene repression such as heterochromatin formation might be a conserved mechanism for stem cell self-renewal.

On the other hand, the Polycomb Group (PcG) complexes are also important for epigenetic gene repression and chromosomal compaction, and components of PcG complexes have been in stem cell biology as well^35^,^36^. Studies have suggested that different PcG proteins may have specific functions in different types of stem cells. One of the major repressive marks in mammalian embryonic stem cells, H3K27 methylation, is catalyzed by a methyl transferase, EZH2 of Polycomb Repressive Complex 2 (PRC2), with the facility of other PRC2 components including EED, SUZ12 and RbAp46/48^37^,^38^. Recent studies have identified crosstalk between H3K9 and K27 methylation: the central components of PRC2, EZH2 and SUZ12, are required for HP1α stability; and binding of HP1α/β/γ to H3K9me3 is greatly enhanced in presence of H3K27me3, indicating a highly interactive relationship between heterochromatin components and polycomb complex^39^. Further exploration of this interaction in pluripotent stem cells, known to depend on chromatin based silencing of developmental gene expression, will be informative for understanding epigenetic mechanisms of stem cell maintenance.

## Methods

### Fly stocks and Genetics

All crosses were carried out at 25 °C on standard cornmeal/agar medium unless otherwise specified. Fly stocks of *hop*^*25*^, *Su(var)205*^*05*^, *Su(var)205*^*04*^, *Su(var)3-9*^*2*^, *nanos-Gal4*, and *hsp70-flp; Act5C* > *y*^*+*^ > *Gal4 UAS-GFP/CyO, and FRT82B, ubq-GFP* were from the Bloomington *Drosophila* Stock Center (Bloomington, IN). RNAi lines of *UAS-HP1 RNAi,* and *UAS-Su(var)3-9 RNAi* were from Vienna *Drosophila* RNAi Center (VDRC; Vienna,Austria). Fly stocks of *esg-GFP* (YB0232; L. Cooley), *Su(var)3-9*^*6*^ and *Su(var)3-9*^*17*^ (Gary Karpen), and *bam-GFP* (S. DiNardo) were generous gifts. *UAS-HP1* was constructed by inserting a *Drosophila* HP1 cDNA into *Drosophila* transformation vector pUAST. Standard techniques were used to obtain transgenic flies.

To express UAS-Gene in random clones using the “Flp-out” method^40^, *hsp70-flp; Actin* > *y*^*+*^ > *Gal4 UAS-GFP* flies were crossed to *UAS-transgene* flies, and the progeny were heat-shocked for 1 h at 37 °C. To generate GFP-marked *Su(var)3-9* loss-of-function clones by the FLP/FRT-mediated methods, we crossed *hsp70-flp; FRT*^*82B*^ *Su(var)3-9*^*2*^*/TM3* females to males of *hsp70-flp; FRT*^*82B*^ *ubiq-GFP* to produce “twin-spot” clones^41^. The progeny were heat-shocked at 37 °C for 2 hrs at indicated developmental stages and examined at indicated times after clone induction.

### Immunofluorescence and Western blotting

Mouse monoclonal anti-HP1 (C1A9; 1:50), anti-FasIII (7G10; 1:200; for hub cells), anti-α-spectrin (3A9; 1:10; for fusome) were from Developmental Studies Hybridoma Bank (University of Iowa). Rabbit antibodies against histone H3 (1:1000), H3K4me3 (1:500), and H3K9me3 (1:250) were from Upstate Biotechnology. These antibodies and rabbit anti-Vasa (1:1000; generous gifts from Ruth Lehmann) were used as primary antibodies and fluorescent (Molecular Probes) or HRP conjugated secondary antibodies were used in whole-mount immunostaining or Western blotting, respectively. Tissues were fixed in 4% paraformaldehyde and 0.3% Triton-X. Stained tissues were photographed with a Leica confocal microscope or a Zeiss epifluorescence microscope. Images were cropped and minimally processed with Adobe Photoshop.

### Quantitative Real-Time PCR

Testes were dissected from individual 2-day old males of appropriate genotypes and were severed into tip and body regions in ice-cold Schneider medium. Total RNA was isolated using TRIzol (Invitrogen) according to the manufacturer’s instructions. The first strand complementary DNA (cDNA) was generated from 1 pair of testis-equivalent of purified total RNA using Superscript III reverse transcriptase (Invitrogen) and oligo(dT)12-18. The cDNA was used as template for qPCR analysis using SYBR green based detection on a BioRad iCycler. Reactions were carried out in triplicate, and melting curves were examined to ensure single products. Results were quantified using the “delta-delta Ct” method to normalize to rp49 transcript levels and to control genotypes. Data shown are averages and standard deviations from at least three independent experiments. The following primer pairs were used.

rp49: TCCTACCAGCTTCAAGATGAC, CACGTTGTGCACCAGGAACT

bam: CCAATCGCGCAGACCAATTAGCAA, CGAGTGTGACAAGTTGCTTAAGGG

Vasa: CCCAAATGAACATAGGAGCGATCC, TTTCATCCGCATCAGCTGGTACCA

esg: TACCCATCATCACCATGCGCCTAT, TCCCGGCTGGCTAGTGTTTAGATT

Ubiq: CGTTCTCAATGGTATCGGATGGCT, CACTCTGTCCGACTACAACATCCA

## Additional Information

**How to cite this article**: Xing, Y. and Li, W. X. Heterochromatin components in germline stem cell maintenance. *Sci. Rep.* **5**, 17463; doi: 10.1038/srep17463 (2015).

## Supplementary Material

Supplementary Information

## Figures and Tables

**Table 1:** *Su(var)3-9* is essential for GSC self-renewal.

*Su(var)3-9*^*2*^ mutant clones (GFP^–^)			
Days after Induction	Clone Genotype	Testes with GSCclones/testes scored	testes scored
2	*Wild type*	24/42 (57%)	8/42 (19%)
	*Su(var)3-92*	15/48 (31%)	19/48 (40%)
5	*Wild type*	24/44 (55%)	9/44 (20%)
	*Su(var)3-92*	0/42 (0%)	13/42 (31%)
7	*Wild type*	31/53 (58%)	4/53 (7.5%)
	*Su(var)3-92*	0/30 (0%)	6/30 (20%)
***Su(var)3-9 RNAi* clones (GFP^+^)**			
Days after Induction	Clone Genotype	Testes with GSCclones/testes scored	Testes with non-GSC clones/testes scored
2	*Wild type*	62/67 (92%)	5/67 (7.5%)
	*Su(var)3-9 RNAi*	4/74 (5.4%)	44/74 (59%)
5	*Wild type*	44/51 (81%)	6/51 (12%)
	*Su(var)3-9 RNAi*	3/65 (4.6%)	18/65 (28%)
7	*Wild type*	35/46 (76%)	10/46 (22%)
	*Su(var)3-9 RNAi*	1/27 (3.7%)	5/27 (19%)

Mutant clones homozygous for *Su(var)3-9*^*2*^ (loss-of-function allele) or wild-type (control) were identified as GFP^–^ cells. Knock-down clones expressing *Su(var)3-9 RNAi* or wild-type control were identified as GFP+ cells. Testies with one or more mutant clones at the GSC position were counted as “testes with GCS clones”.
