# Supplementary material for: Heterochromatin components in germline stem cell maintenance
Source: Sci Rep. 2015 Dec 2;5:17463. doi: 10.1038/srep17463 (PMC4667240; doi:10.1038/srep17463)
Supplement: Supplementary Information [file srep17463-s1.pdf]

Supplementary Information

Heterochromatin components in germline stem cell maintenance

Yalan Xing[1,a] and Willis X. Li[1, 2]

[1] Department of Biomedical Genetics, University of Rochester Medical Center, Rochester, NY 14620

[2] Department of Medicine, University of California San Diego, La Jolla, CA 92093.

[a] Current Address: Department of Biochemistry, Institute for Stem Cell and Regenerative Medicine, University of Washington, Seattle, WA 98109

Table S1. Requirement for HP1 in GSC maintenance

Days after clone induction	Clone genotype	Testes with GSC clones/testes scored
1	WT control	15/18 (83%)
	Su(var)205^{5}	2/20 (10%)
2	WT control	36/37 (83%)
	Su(var)205^{5}	3/16 (19%)
5	WT control	8/11 (73%)
	Su(var)205^{5}	1/14 (7.1%)
7	WT control	27/31 (87%)
	Su(var)205^{5}	0/38 (0%)

"Twin-spot" clones homozygous for *Su(var)205*^{05} (loss-of-function allele) or wild-type (control) were identified as GFP^{-} cells. Testes with identifiable clones were scored. Those with at least one clone at GSC position were counted as "testes with GCS clone".

Figure S1

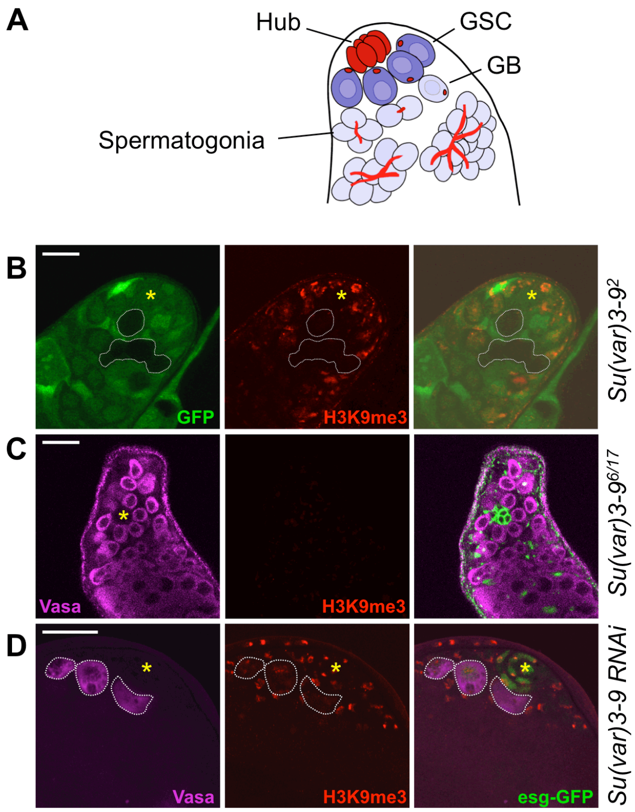

Fig. S1. Su(var)3-9 mutation or RNAi knock-down reduces heterochromatin

Scale bar = 20 µm

(A) Schematic representation of stem cell differentiation in the *Drosophila* testis. Germline stem cells (GSCs; blue) are adjacent to hub cells (red). GSCs differentiate to give rise to gonialblasts (GBs; light blue), which divide to give rise to spermatogonia. See text for more detail.

(B) Testes from *hsp70-flp; FRT*^{82B} *ubi-GFP/FRT*^{82B} *Su(var)3-9*^{2} were dissected 2 days after heat-shock induction of somatic recombination and were stained for H3mK9 (red). Note that the *Su(var)3-9*^{2} homozygous cells (GFP^{-}; circled by dotted line) have no detectable H3mK9 signal.

(C) Testes from 2-day old *Su(var)3-9*$^{6/17}$ males were stained for Vasa (magenta) and H3mK9 (red), and the hub and fusome (green). Note that the testis lacks H3mK9 signal.

(D) Testes from 2-day old wild-type males expressing esg-GFP, nos-Gal4, and UAS-Su(var)3-9 RNAi were stained for Vasa (magenta) and H3mK9 (red). Note that the remaining Vasa+ cells (circled by dotted lines) exhibit much reduced H3mK9 signal compared with the rest of testis area.

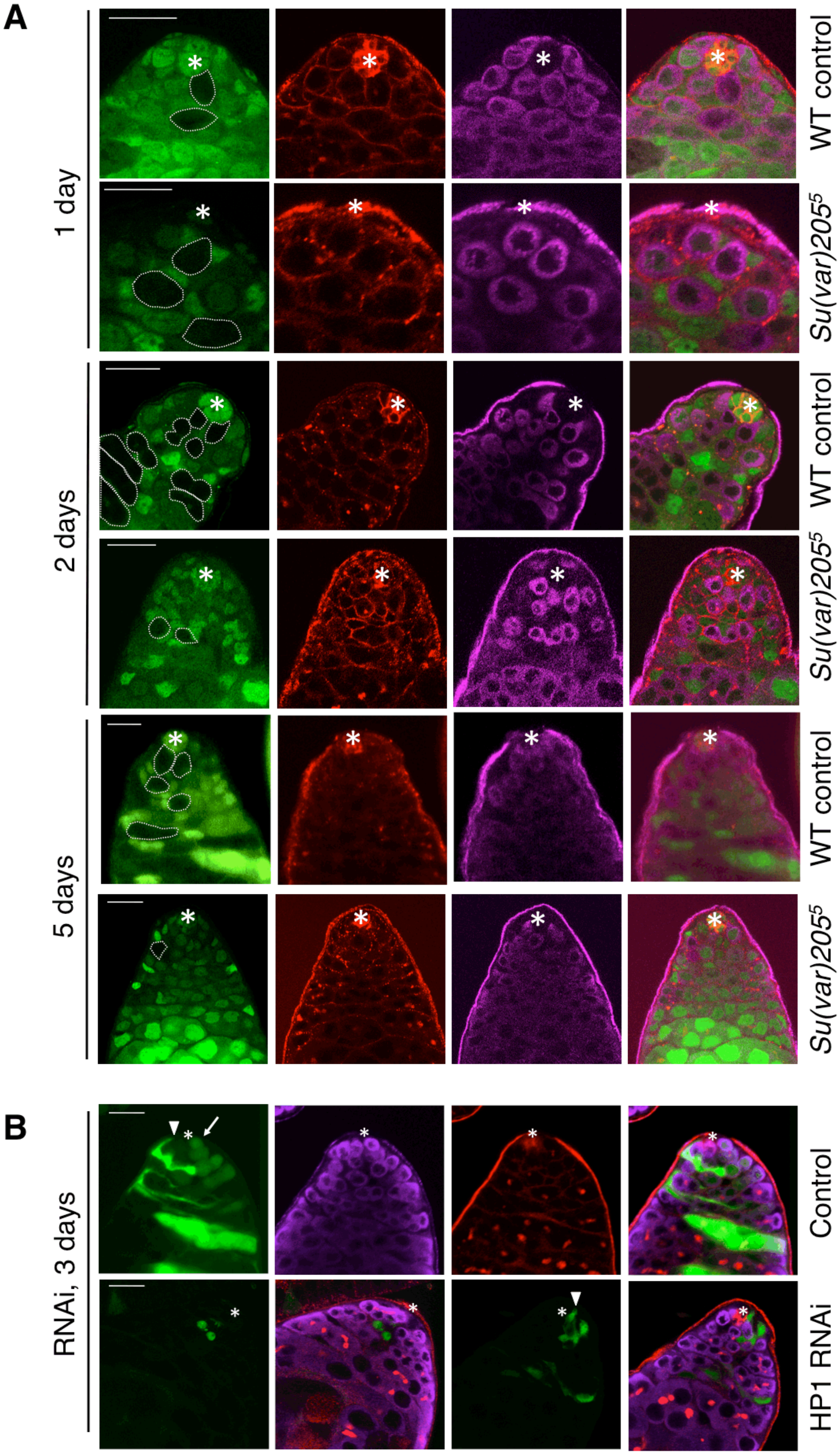

A
B
1 day
2 days
5 days
RNAi, 3 days
WT control
Su(var)205[5]
WT control
Su(var)205[5]
WT control
Su(var)205[5]
Control
HP1 RNAi

Figure S2. Effects of loss of HP1 on GSC maintenance

Testes from male flies that had been subjected to induction of marked clones for loss-of-function of HP1 (GFP^{-}) (**a**) or over-expression of HP1 RNAi (GFP+) (**b**) were immunostained for Vasa (magenta cytoplasmic staining) and for fusomes and hub cells (both red). Asterisk indicates the position of the hub. Scale bar=20 μm.

(A) Testes were dissected after 1, 2, or 5 days after clone induction. Wild-type control and *Su(var)205*^{5} homozygous mutant clonal cells were marked by the absence of GFP (lack of green). Note that wild-type control clones were found at GSC position (next to the hub), and that *Su(var)205*^{5} homozygous mutant clones were found at he GSC position only 1 day after clone induction, but not after 2 or more days.

(B) In control testes (which did not express HP1 RNAi) 3 days after clone induction, clonal GFP+ cells were found in both the germline (round Vasa+ cells) and somatic (elongated Vasa-minus cells) lineages. Arrow marks a GSC, and arrowhead a CPC. Three days after HP1 RNAi was expressed, no HP1 RNAi-expressing (GFP+) GSCs were observed next to the hub. GFP+ germline cells (round green cells in left panels) were found away from the hub, and they now cease to express Vasa. GFP+ somatic cells (long $Vasa^{-}$ cells in right panels), however, were still observed.

Figure S3

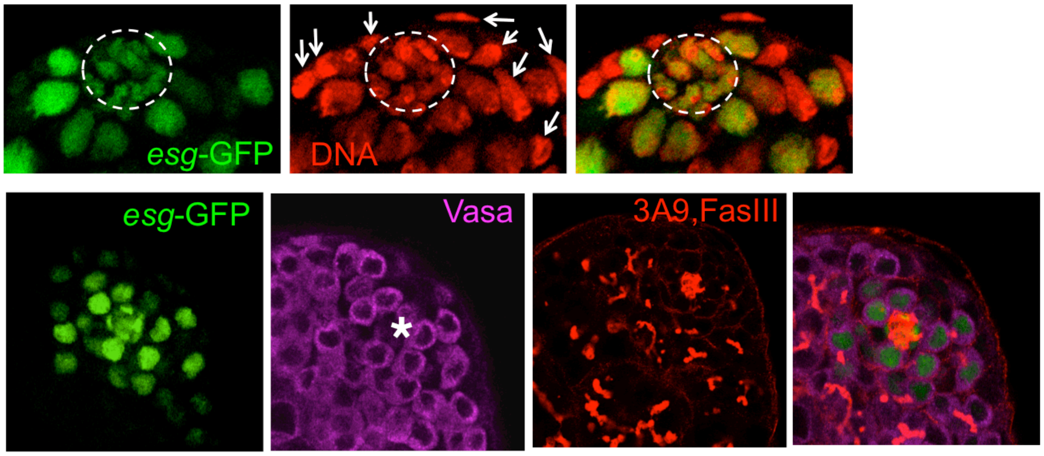

Fig. S3. Expression and localization of esg-GFP

Testes from 2-day old *Drosophila* wild-type males expressing esg-GFP were stained for DNA (red; upper), or Vasa (magenta; lower), or fusome and hub cells (red; lower). Hub cells are circled with dashed line (upper) or marked by asterisk (lower). Arrows point to CySCs. Note that esg-GFP+ cells colocalize with Vasa+ cells, but not with CySCs (Vasa^{-} cells).
